# Web-Based Lifestyle Interventions for Survivors of Cancer: Usability Study

**DOI:** 10.2196/30974

**Published:** 2022-02-21

**Authors:** Victoria Williams, Nashira Brown, Justin Xavier Moore, David Farrell, Suzanne Perumean-Chaney, Erica Schleicher, Kevin Fontaine, Wendy Demark-Wahnefried, Dori Pekmezi

**Affiliations:** 1 Department of Health Behavior University of Alabama at Birmingham Birmingham, AL United States; 2 Department of Population Health Sciences Augusta University Augusta, GA United States; 3 People's Design Inc. Durham, NC United States; 4 Department of Biostatistics University of Alabama at Birmingham Birmingham, AL United States; 5 Department of Nutritional Sciences University of Alabama at Birmingham Birmingham, AL United States

**Keywords:** cancer survivors, diet, exercise, lifestyle, internet, physical activity, web-based, website, weight management, digital health, cancer, online health

## Abstract

**Background:**

Internet-based lifestyle programs are increasingly being used to deliver health behavior change interventions to survivors of cancer. However, little is known about website use in this population or its association with healthy lifestyle changes.

**Objective:**

The aim of this study is to describe lifestyle intervention website use (log-ins, time on website, and page views) among survivors of cancer and patterns of use by participant characteristics. In addition, associations were explored between website use and changes in healthy lifestyle knowledge and practice.

**Methods:**

A total of 35 survivors of cancer were recruited between August 2017 and 2018 to participate in a 2-week, single-arm pilot test of the SurvivorSHINE lifestyle intervention website. Knowledge and practices related to healthy diet and physical activity behaviors were measured at baseline and follow-up. Website use (eg, time spent on the website, frequency of log-ins, and page views) were collected from the SurvivorSHINE administrative site during the intervention period. Patterns of use were examined by participants’ gender and race. Correlations between website use and changes in healthy lifestyle knowledge, physical activity, diet, and weight were explored. Mann–Whitney *U* tests were used to compare demographic factors on website use.

**Results:**

Participants logged into the SurvivorSHINE intervention website an average of 3.2 (SD 2) times over the 2-week period and spent a total average of 94 (SD 56) minutes viewing the website during the intervention. Examining website activity, 1905 page views were logged. The *User Profile* (344 page views) and *Home* sections (301 page views) were the most frequently visited components. No associations were observed between the frequency of log-ins or the total time on the website, improvements in knowledge related to healthy lifestyles, or changes in body weight or dietary intake. However, the total time on the website was positively correlated with improvements in accelerometer-measured physical activity (*r*=0.74; *P*=.02) and self-reported physical activity (*r*=0.35; *P*=.04).

**Conclusions:**

Survivors of cancer demonstrated clear interest in a diet and exercise intervention website, as evidenced by their frequency of log-ins, page views on numerous features, and total viewing time. Moreover, increased website use was correlated with improvements in physical activity.

## Introduction

### Background

Emerging evidence shows that web-delivered interventions can improve diet [1-3], physical activity [1-16], and weight management [10,17,18] in survivors of cancer [19-21]. Previous studies have reported positive associations between the lifestyle intervention website use by survivors of cancer and improvements in diet (ie, vegetable consumption) [2], physical activity (ie, aerobic and resistance-based) [13,22], and behavioral outcomes [3,23,24]. However, to date, most of these studies have been conducted predominantly on survivors of breast or prostate cancer who are White [25]. As survivors of cancer are growing in number and becoming more diverse by cancer type, age, and race [26], this study fills a critical research gap by investigating lifestyle intervention website use in a more heterogeneous sample in which racial minorities and various cancer types beyond the breast and prostate are represented. Moreover, cancer is a disease associated with aging; however, extant studies have primarily enrolled younger survivors of cancer [24,27]. Thus, conducting more web-delivered lifestyle interventions within an older and more diverse cancer survivor population provides a more thorough understanding of how to better design these interventions to engage survivors of a variety of cancers to make a larger impact.

Most survivors of cancer in the United States are older adults (>65 years), as the median age of cancer diagnosis in the United States is 66 years [28]. Although older adults have lagged behind younger adults in internet use in the past [29], more recent studies have found that older adults are the fastest-growing segment of internet users and the digital divide is closing [30]. However, disparities still exist among older adults with regard to internet use, with affluent, well-educated adults using the internet more than other subgroups [31]. However, initial concerns that internet-delivered interventions might not be appropriate for survivors of cancer, as most are older adults appear unfounded [29]. Questions remain about which segments of survivors are most suited for web-based healthy lifestyle interventions, how such programs work, and whether website use is associated with health benefits among survivors of cancer. Given the recent increases in survivors of cancer as well as technology adoption, a better understanding of healthy lifestyle website use patterns and outcomes among older survivors of cancer would help inform future optimization of the experience and related outcomes.

### Objectives

The purpose of this research is to describe the use (log-ins, time on the website, and page views) of a web-based healthy lifestyle program, SurvivorSHINE [32], in a pilot study for adult survivors of cancer. Furthermore, we examined patterns of use by participant characteristics (gender and race) and associations between website use and changes in knowledge, body weight, diet, and physical activity. It was hypothesized that (1) upon log-in to SurvivorSHINE, survivors of cancer will visit key features, such as body weight, diet, and physical activity sections of the website, similar to use rates found in past studies with survivors of breast or prostate cancers who are predominantly White [19-21]; (2) survivors of cancer with higher website use will have greater improvements in healthy lifestyle knowledge, and exhibit changes in physical activity, diet quality, and weight, given positive associations between website use and behavior change (increased physical activity) in previous research [33]; and (3) survivors who are non-Hispanic White and men will have higher website use compared with survivors who are non-Hispanic Black and women, given Pew Research Center data indicating particularly high computer and internet use rates in these groups [34,35].

## Methods

### Research Design

Data for this study were collected from survivors of cancer participating in a single-arm pilot study of the SurvivorSHINE lifestyle intervention website. Initial findings have already supported feasibility, acceptability, and behavior change (improvements in physical activity, healthy lifestyle knowledge, etc) in response to SurvivorSHINE [32]. The current analyses focus on website metrics (time spent on websites, log-ins, and page visits) over the 2 weeks of use and their relationship with demographics at baseline and lifestyle factors (healthy lifestyle knowledge, body weight, diet, and physical activity) at baseline and after the intervention.

### Participants

Participants were survivors of cancers with >80% 5-year survival (eg, cancers of the breast, prostate, and thyroid). The eligibility criteria were (1) non-Hispanic White and non-Hispanic Black adults, (2) residence within a 20-mile radius of the University of Alabama at Birmingham, (3) self-reported internet access and regular use, and (4) ability to speak and read English. Recruitment was completed using the following methods: (1) ascertaining patients with cancer from the University of Alabama at Birmingham Cancer Registry and sending a letter of invitation, (2) contacting local cancer survivor support groups and related community organizations, (3) local news advertisements, and (4) word of mouth.

### Protocol Overview

Participants completed the assessments (anthropometrics, healthy lifestyle knowledge, and current physical activity and dietary practices) in person with study staff, whereas screening was conducted by telephone. Participants were then given access to the SurvivorSHINE website and encouraged to review and use all of its components (ie, update user profile and visit different sections of the website) as frequently as possible (ideally, daily) over the 2-week website use period. Participants were instructed to create a profile using a username or personal email along with a password. Moreover, they were directed to access the website via their personal profiles each time. At the completion of the intervention period, participants completed follow-up assessments and exit interviews in person [32]. Full details of the trial design, participants, protocol, measures, and intervention are described in a previous report [32] but are briefly outlined below.

### Intervention Website

SurvivorSHINE is a theoretically-driven (social cognitive theory) [32], web-delivered intervention promoting physical activity, a healthy diet, and weight management among survivors of cancer. SurvivorSHINE was largely adapted from the written materials that were used in the Reach-out to Enhance Wellness intervention, which demonstrated proven efficacy in improving diet quality and physical activity, as well as body weight and physical functioning among 641 older survivors of cancer [36]. The website was designed using input obtained from 4 sets of focus groups stratified by gender (male or female) and race (White or Black) [32]. It includes six main sections: *My Profile*, *Home Page*, *Healthy Weight*, *Healthy Eating*, *Exercise*, and *News You Can Use*. For this study, users created an account and entered data on their demographics and cancer type in the *My Profile* section to guide the personal tailoring of the diet, weight management, and exercise information. Direct links for the *Healthy Eating*, *Healthy Weight*, *Exercise*, and *News You Can Use* sections of the website were provided on the *Home Page*. The *Healthy Eating* section generated personalized feedback on the users’ diet and information on a healthy diet, fast food, serving sizes, and tips to promote healthy eating behavior. Moreover, specific guidance was given to diet-related domains that corresponded with the American Cancer Society guidelines for survivors of cancer [37] and encouraged to achieve the following dietary goals for vegetables and fruits (5 daily servings), whole grains (≥50% of grain consumption), added sugar (≤6 teaspoon per day for women and ≤9 teaspoon per day for men), red and processed meat (<0.51 kg per week), saturated fat (<10% of total calories), and alcohol consumption (to achieve an eventual goal of ≤1 drink per day for women and ≤2 drinks per day for men).

Individualized feedback was also provided in the *Exercise* section based on the physical activity information users entered in their profile. More specifically, participants were provided with tailored recommendations based on physical activity information input in the user profile. Incremental increases in the frequency and duration of endurance exercise resulted in a net increase of 30 minutes per week (ie, 150 minutes per week) and frequency of strength training (ie, 2-3 times per week) to meet the American Cancer Society guidelines for survivors of cancer [33]. Moreover, this section highlights leg strengthening exercises, the benefits of exercise, the use of pedometers and accelerometers, the importance of setting SMART (specific, measurable, attainable, relevant, and timely) goals [38], and a calorie burning guide. Feedback on users’ weight was provided in the *Healthy Weight* section, which featured a BMI calculator, calorie calculator, and sample meal plans. Finally, easy-to-read summaries of recent cancer-related healthy lifestyle research were provided in the *News You Can Use* section; this section was highly endorsed by focus group participants [32].

### Measures

Demographic information assessed at baseline included age, gender, race or ethnicity, the highest level of educational attainment, marital status, current employment, and income. Healthy lifestyle knowledge, physical activity, diet, and weight were measured at baseline and follow-up. Survivors’ healthy lifestyle knowledge was evaluated using a 10-item questionnaire about the American Cancer Society recommendations for diet, physical activity, and weight management [32]. Physical activity was assessed subjectively using the Godin Leisure-Time Exercise Questionnaire and assessed objectively using an accelerometer [39]. The Godin Leisure-Time Exercise Questionnaire has acceptable reliability, internal consistency, and similar validity with more objective measures of physical activity levels [40-42]. ActiGraph accelerometers (ActiGraph GT3X+) were used to collect objective measures of physical activity and have been validated using heart rate telemetry [43] and total energy expenditure [44]. The ActiGraph protocol used has been previously reported [32]; in brief, the minimum threshold for moderate intensity was set at 1952 counts per minute [45]. In addition, the minimum valid wear time was set at 4 days for at least 600 minutes of wear. The Automated Self-Administered 24-Hour Dietary Assessment, a validated web-based tool [46], was used to capture dietary recalls for 2 days, including 1 weekday and 1 weekend day. The Automated Self-Administered 24-Hour Dietary Assessment tool generates a nutrient analysis of beverages and food consumed with variables of interest, including total kilocalories, total fat, saturated fat, added sugar, alcohol, and servings of vegetables and fruits, whole grains, and red meat. Weight was measured without shoes and in light clothing using the Health O Meter Floor Scale (894KLTEA).

Website use was measured by use statistics of time spent on the website, frequency of log-ins, and page views. Log-ins, or the number of times a user signs in, were collected each time a user signed in using a username or email address and password. Page views, or the number of times a user visits a page of the website, were determined based on website logs that indicated pages users visited (eg, navigating to the *Healthy Eating* section of the website required users to visit a specific link). Website use data and logs were obtained from the administrative site of SurvivorSHINE, which contained time-stamped activity records for participant accounts (eg, participant 27 clicked the *Healthy Eating* section at 2:34 PM on December 15, 2019) [32]. The website use data were connected to trial participants through website usernames and passwords associated with each study identifier. Although the website was publicly available, only website use data connected to actual trial participants were included in the analyses.

### Data Collection and Analyses

Although this study was exploratory, power calculations suggest that given a sample size of 35, this study has 99% power to detect strong correlations of 0.7 and 68.1% power to detect moderate correlations of 0.4. Power analyses were computed using SAS (version 9.4; SAS Institute Inc). The analyses included descriptive statistics and frequencies of website use. Owing to the nonnormality of data and small sample size, Spearman rank correlations examined the relationships between website use and healthy behavior knowledge, as well as behavior change (physical activity, dietary intake, and weight). Mann–Whitney *U* tests were used to compare demographic factors on website use. All analyses were performed using SPSS (version 25; IBM Corp). For this study, data from participants who completed both baseline and follow-up measures were included (35/41,85%).

### Ethical Considerations

The protocol was approved by the University of Alabama at Birmingham Institutional Review Board (IRB-140428003), and signed informed consent was obtained from all participants.

## Results

### Participants

The participants were survivors of many types of cancers, but breast and prostate cancers were the most common diagnoses. The average age of the sample was 62.1 (SD 11.9) years, and most were women (19/35, 54%), non-Hispanic White (22/35, 63%), married (24/35, 69%), and retired (19/35, 54%). Income and educational levels were higher than state averages, with more than half of the sample reporting annual household incomes surpassing US $50,536 and baccalaureate or higher degrees [47] (Table 1).

**Table 1:** Sample characteristics (N=35).

Variable			Values
Age (years), mean (SD)			62.1 (11.9)
**Gender, n (%)**			
	Female	19 (54)	
**Race, n (%)**			
	Black	13 (37)	
	White	22 (63)	
**Cancer type^a^, n (%)**			
	Breast	16 (46)	
	Prostate	12 (34)	
	Myeloma	2 (6)	
	Skin	2 (6)	
	Thyroid	2 (6)	
	Head and neck	2 (6)	
**Marital status, n (%)**			
	Married or civil union	24 (69)	
	Widowed	5 (14)	
	Single, never married	3 (9)	
	Divorced	3 (9)	
**Education, n (%)**			
	High-school degree of equivalent (eg, General Educational Development)	4 (11)	
	Some college but no degree	4 (11)	
	Associate degree	8 (23)	
	Bachelor’s degree	11 (31)	
	Graduate degree	8 (23)	
**Income (US $), n (%)**			
	≤49,999	13 (37)	
	50,000-124,999	10 (29)	
	≥125,000	9 (26)	
	Refused	3 (9)	
**Employment, n (%)**			
	Employed, working full-time	9 (26)	
	Employed, working part-time	3 (9)	
	Disabled, not able to work	4 (11)	
	Retired	19 (54)	

^a^Participants may select multiple cancers.

### Intervention Website Use

On average, participants logged 3.2 (SD 2) times and spent an average of 31 (SD 16) minutes per log-in. Thus, the total time spent on the website during the 2-week period was 94 (SD 56) minutes. On average, users visited 1905 different areas of the website, as evidenced by the 1905 page views (Table 2). Over the 2-week period, page views ranged from 2 to 153 page views per user. Participants averaged a total of 57.7 (SD 30.7) page views per person and 20 (SD 10.2) page views per log-in. Overall, the initial *User Profile* (344 page views) and the *Home* sections (301 page views) were the most visited components of SurvivorSHINE (Table 2, column 2). The *News You Can Use* section was the least visited section with only 91 overall total page views. When these data were examined at the individual level (page views per user), a more thorough website exploration was found. For example, participants also frequently viewed the *Healthy Eating*, *Healthy Weight*, *Exercise*, and *News You Can Use* sections (32 user page views; Table 2, column 3). Overall, participants spent the most time on the *User Profile* section updating personal exercise, weight, and eating information (62,912 seconds). On average, the most time per participant was spent on the *User Profile* (mean 183 seconds, SD 173 seconds), in the *Healthy Weight* section viewing sample meal plans (mean 110 seconds, SD 96.3 seconds), and learning about accelerometers and pedometers in the *Exercise* section (mean 89 seconds, SD 71.2 seconds).

**Table 2:** SurvivorSHINE key components accessed by participants (N=1905).

SurvivorSHINE website component		Total page views (page views ÷ total page views; N=1905), n (%)	User page views (page views ÷ total users; N=35), n (%)	Page views per user, median (IQR)	Total time spent on component in seconds, n	Average time spent on component per user in seconds (total time on component ÷ total users; N=35), mean (SD)
User profile		344 (18)	32 (97)	8 (9)	62,912	183 (173)
Home		301 (16)	31 (94)	9 (7)	9237	31 (33.1)
**Healthy eating**		196 (10)	32 (97)	4 (6)	8972	46 (44.6)
	Vegetables and fruits	51 (3)	15 (46)	1 (0)	861	32 (21.2)
	Benefits of a healthy diet	26 (1)	16 (49)	2 (0)	1135	44 (41.1)
	Fats and fast food	24 (1)	17 (52)	1 (1)	1304	54 (16)
	Sugar	21 (1)	19 (58)	1 (1)	1084	52 (24.2)
	Meat	14 (0.7)	12 (36)	1 (0)	685	49 (34.3)
	Whole grains	10 (0.5)	9 (27)	1 (0)	418	42 (34)
	Alcohol	11 (0.6)	10 (30)	1 (0)	234	21 (12.1)
**Healthy weight**		192 (10)	32 (97)	6 (5)	6383	33 (68.1)
	Calorie calculator	54 (3)	25 (76)	2 (1)	2160	40 (24.4)
	BMI calculator	47 (3)	25 (76)	2 (1)	4142	86 (199)
	Sample meal plans	39 (2)	18 (55)	3 (1)	4286	110 (96.3)
	Benefits of healthy weight	31 (2)	16 (49)	2 (0)	1276	41 (28)
	Common questions	24 (1)	14 (42)	1 (0)	1420	59 (54)
**Exercise**		124 (7)	32 (97)	3 (4)	6717	54 (63)
	Resistance training exercises	38 (2)	23 (70)	2 (1)	1399	37 (23)
	Benefits of exercise	29 (2)	18 (55)	1 (1)	1276	41 (28)
	Calorie burning guide	28 (2)	19 (58)	1 (1)	980	35 (14)
	Accelerometers and pedometers	28 (2)	21 (64)	1 (1)	2490	89 (71.2)
	SMART^a^ goals	27 (1)	20 (61)	1 (1)	873	32 (14)
News you can use		91 (5)	32 (97)	2 (3)	6984	77 (67.1)

^a^SMART: specific, measurable, attainable, relevant, and timely.

### Behavior and Use Patterns

Survivors who are men and non-Hispanic White spent relatively more time on the website than survivors who are women and non-Hispanic Black. However, the time spent on SurvivorSHINE and website page views did not significantly differ by race or gender (Table 3). The frequency of log-ins was also not significantly different by subgroup; however, survivors who are non-Hispanic White had somewhat more log-ins than survivors who are non-Hispanic Black (*P*=.06).

The total time spent on the website was positively and significantly correlated with changes in self-reported and measured physical activity (Table 4). The frequency of log-ins was correlated with changes in measured physical activity but not self-reported. The total time on the website, frequency of log-ins, and page views were not correlated with changes in healthy lifestyle knowledge, weight, and diet.

**Table 3:** Mean rank log-ins, page views, and total time spent on SurvivorSHINE website by race and gender.

Variables	Female (n=17)	Male (n=16)	Mann–Whitney *U* test		Black (n=13)	White (n=20)	Mann–Whitney *U* test	
			*Z* value	*P* value			*Z* value	*P* value
Page views	16.5	17.6	0.32	.76	14.2	18.8	1.35	.18
Log-ins	16	18.1	0.65	.53	13.1	19.6	1.94	.06
Total time (minutes)	16.8	17.2	0.11	.93	14.7	18.5	1.12	.27

**Table 4:** The correlation between website use and posttest diet, exercise, and healthy lifestyle knowledge measures.

Measures		Total time		Log-ins			Page views	
		r_s_	*P* value	r_s_	*P* value	r_s_		*P* value
Physical activity (measured)		0.73	.02	0.73	.02	.48		.19
Physical activity (self-reported)		−0.35	.049	−0.15	.41	−0.12		.51
Knowledge		0.04	.81	0.08	.66	−0.02		.91
Weight		0.15	.39	−0.01	.93	0.11		.54
**Diet**								
	Total kilocalories	0.03	.90	0.11	.64	−0.03		.90
	Saturated fat	0.07	.76	0.16	.49	−0.09		.68
	Meat	−0.11	.62	0.10	.65	0.22		.34
	Alcohol	−0.06	.80	−0.07	.75	0.02		.92
	Fruit	0.17	.46	0.16	.49	0.16		.49
	Vegetables	−0.001	.99	−0.21	.37	−0.27		.23

## Discussion

### Principal Findings

Overall, these findings indicate that survivors of cancer are willing to use and devote time to a healthy lifestyle website intervention. During the intervention, participants logged onto the SurvivorSHINE intervention website an average of 3.2 times and spent an average of 94 minutes viewing the website content over the 2-week intervention period. The *User Profile* and *Home* sections were the most visited components of the website. Our results found a positive correlation between the total time on the website and improvements in accelerometer-measured physical activity and self-reported physical activity. No other associations were observed between website use and improvements in knowledge related to healthy lifestyles, body weight, or dietary intake.

The frequency of website log-ins (an average of 3 times over 2 weeks, 1.5 log-ins per week) is consistent with other web-based lifestyle interventions among survivors of cancer and is comparable with the 5.3 times over 6 weeks reported by Bantum et al [4] and 10.3 times over 9 weeks reported by Forbes et al [5] (ie, approximately 1 log-in per week). The study participants also spent an average of 31 minutes per log-in while visiting the SurvivorSHINE website, which is more than the 12 and 11.3 minutes per log-in reported by 2 other healthy lifestyle interventions for survivors of breast cancer [13,22]. The survivors of cancer in this study were highly motivated to use the SurvivorSHINE website likely for its tailored, easy-to-read, and readily accessible healthy lifestyle information and resources [32].

This study also highlights web content on healthy lifestyle behaviors that may be most appealing to the survivors. The user profile was the most visited (number of page views and time spent) component of the website. We speculate that this was the most visited component as participants were encouraged to frequently update their user profile to ensure relevant tailored feedback and information on diet, exercise, and weight loss were received [32]. Comparably, Chen et al [48] reported the most frequently visited page among survivors of breast cancer was *setting goals* where users received personalized feedback based on individual goals and activities. Of the *Healthy Eating*, *Healthy Weight*, and *Exercise* sections, participants spent the most time in the *Healthy Eating* section. Moreover, the *Healthy Eating* section was the most visited lifestyle section of the website; however, changes in diet, as noted in a previous report, were fairly minimal [32], as were changes in weight. However, it must be borne in mind that the study period was only 2 weeks in duration; thus, the −0.3 kg change in body weight noted in the previous report may be clinically significant but did not produce a statistically significant correlation with website use. Similarly, changes in dietary intake may not correlate highly with website use, as the 2-week study period may not have allowed enough time to purchase healthier foods, such as fruits and vegetables, and procure substitutes for red meat. However, despite the *Exercise* section being the least visited of the 3 main health behavior change sections of the website, significant improvements in physical activity were noted as well as significant correlations with website use.

The duration and frequency of the log-ins were positively associated with increased physical activity. Therefore, survivors of cancer who frequently logged on and spent substantial time on SurvivorSHINE were more physically active. Similar associations between website use and increased physical activity have also been reported in previous studies of older adults and Latinas. For example, a previous randomized controlled trial examining a web-based intervention to promote physical activity in sedentary older adults [49] found that higher levels of program use (website visits and total time) were associated with greater changes in physical activity [33,49]. Moreover, a recent web-based computer-tailored physical activity trial in older survivors of prostate cancer reported that increased engagement in the physical activity web component was associated with a higher level of moderate to vigorous intensity physical activity at follow-up [50]. Although this study corroborates and extends past findings to survivors of cancer, further investigation of website components that promote healthy lifestyle behaviors among survivors of cancer is needed in a larger population to learn more about the roles of diet and weight components.

This study also sheds light on the subgroups of survivors of cancer who might be more engaged in web-based approaches. The frequency of log-ins, time on the website, and page views did not significantly differ by race and gender, which supports the accessibility and utility of eHealth tools for diverse populations and allays concerns regarding digital divide. However, although a past review found that samples for past web-based lifestyle intervention studies for survivors of cancer were comprised mostly of non-Hispanic White women [21], trends in the current data suggested slightly greater time spent on website and page views in this group; such programs may be particularly appealing to non-Hispanic White women*.* Although there is limited research on web-based physical activity intervention usability in relation to race and gender, a review of web-based lifestyle interventions among survivors of cancer found that most of the participants in studies that recruited various cancer types were non-Hispanic White and women [21]. This is similar to the usability trend found in this study, suggesting that non-Hispanic White women frequently participate in web-based interventions and may be particularly interested in such programs. Thus, further investigation on how to make the website more relevant to survivors of cancer who are men and non-White may be necessary, especially given the association between website use and behavior change.

### Strengths and Limitations

Although minority representation among survivors of cancer was a strength of this study, the sample was still predominately affluent and well educated, which limits the generalizability of these findings to other survivor groups. Another primary weakness was the number of tests conducted and the high probability of a type 1 error. An additional limitation was the possibility that participants could have created more than one account. In fact, 1 participant had 2 accounts (one where they registered with their work email and another account registered with their personal email) but was taken into account, which also could lead to inaccuracies in website use recordings. Moreover, inaccuracies in metrics of time spent on the website could have occurred in situations where users walked away from the computer or navigated to other websites while still logged into their SurvivorSHINE account. However, the available analytics were helpful in providing a rough estimate of website use for this study. Finally, this study was brief in duration and had a small sample size.

As for future directions, findings from this study need to be replicated and further examined in larger, longer-term studies. Qualitative research should be conducted to examine factors that influence the number of log-ins, time spent on the overall website, and specific features of web-based lifestyle interventions for survivors of cancer (eg, determining why the *Healthy Eating* section was visited more than the *Exercise* section).

### Conclusions

The findings of this study provide insights into how survivors of cancer use web-based healthy lifestyle interventions. Diet content was popular, whereas exercise was less so, but perhaps more helpful, as analyses suggest that internet-delivered lifestyle program use was positively associated with increases in physical activity within this population. Website use and patterns were similar across demographic factors, which is promising for the potential reach of web-based lifestyle interventions for various segments of survivors of cancer.

## Abbreviations

SMART: specific, measurable, attainable, relevant, and timely
